# Sensitization to airborne allergens among adults and its impact on allergic symptoms: a population survey in northern Vietnam

**DOI:** 10.1186/2045-7022-4-6

**Published:** 2014-02-10

**Authors:** Hoàng Thị Lâm, Linda Ekerljung, Anders Bjerg, Nguyễn Văn Tường, Bo Lundbäck, Eva Rönmark

**Affiliations:** 1 Department of Allergy & Clinical Immunology, Hanoi Medical University, Hanoi, Vietnam; 2 Department of Allergy and Clinical Immunology, Bachmai Hospital, Hanoi, Vietnam; 3 Department of Internal Medicine/Krefting Research Centre, Sahlgrenska Academy, University of Gothenburg, Gothenburg, Sweden; 4 Department of Scientific Research, Hanoi Medical University, Hanoi, Vietnam; 5 The OLIN Studies, Norrbotten County Council, Luleå, Sweden; 6 Department of Public Health and Clinical Medicine, University of Umeå, Umeå, Sweden

**Keywords:** Allergic sensitization, Asthma, Allergic rhinitis, Storage mite, Vietnam

## Abstract

**Background:**

The knowledge about allergic sensitization and its relationship with clinical symptoms and diseases among adults in South-East Asia is poor. The aim of this study was to investigate the prevalence and pattern of allergic sensitization and the association with asthma and allergic rhinitis in adults in urban and rural Vietnam.

**Methods:**

Among 5,782 responders to a questionnaire survey in northern Vietnam, a random sample was invited to a clinical follow-up and 684 (46%) participated. The methods included a structured interview using a modified GA^2^LEN study questionnaire on symptoms and possible determinants for diseases. Skin prick test (SPT) with ten common airborne indoor and outdoor allergens, lung function test, and methacholine test was performed among subjects ≤60 years of age.

**Results:**

In total, one third of subjects had a positive SPT reaction to at least one allergen, 36.9% of men and 31.0% of women (n.s.). The most common sensitizer was the storage mite *B. tropicalis* (men 27.7%; women 18.7%) followed by house dust mite *D. pteronyssinus* (men 16.5%; women 10.6%), and *D. farinae* (men 15.3%; women 6.3%), and cockroach (men 16.5%; women 10.2%). Sensitization to all major allergens were significantly more common among men and among subjects ≤45 years compared with women and subjects >45 years, respectively. The prevalence of sensitization to animals, pollen and molds were low. The majority of cockroach-sensitized subjects were also sensitized to mites. Sensitization to any allergen and all major allergens were significantly associated with rhinitis, but not with asthma. However, bronchial hyper-reactivity was significantly associated with increasing number of positive SPTs (p = 0.047).

**Conclusions:**

Among adults in northern Vietnam sensitization to mite and cockroach most common in both rural and urban areas. The dominant sensitizer was the storage mite *B. tropicalis*, which should be included in future studies and also in clinical practice, owing to its association with clinical symptoms. As in the Western world allergic sensitization was associated with rhinitis and bronchial hyper-reactivity. The lack of association with asthma in South-East Asia needs to be studied further.

## Introduction

In Vietnam, contrast to the westernized world, the prevalence of allergic sensitization and the patterns of sensitization to different allergens have been less investigated. Studies of children have been performed in Singapore, Thailand and Malaysia [1-3], however data from unselected populations is very limited, especially in adults. In adult asthmatics in these areas, house dust mite and cockroach were the main allergens [4,5]. Also in the middle and southern part of Europe, house dust mite is the most common allergen [6]. In temperate areas with a colder climate like Finland and Sweden, furry animals such as cat and dog, together with pollen are the dominant sensitizers [7,8].

In southern Vietnam, population based studies in children and adolescents found a high prevalence of sensitization to house dust mite and cockroach, while sensitization to moulds and pollens was significantly less common [9,10]. This pattern was seen also in the first population based study of sensitization in adults in Northern Vietnam, and we recently presented the crude prevalence measures in a short report [11]. To date, the impact of sensitization on allergic symptoms and conditions in the general adult population in this area has not been studied.

In the current paper we present in-depth analyses of allergic sensitization and its impact on allergic symptoms and diseases. The aim was to characterize sensitization in urban and rural areas in relation to age and gender, and to study the association between allergic sensitization and typical allergic conditions as asthma and rhinitis.

## Material and methods

### Study area and population

The study area included an urban and a rural area in northern Vietnam. The urban area was Hoankiem, an inner-city district comprising the biggest trading centre of Hanoi, with a population density in 2007 of 32,703 inhabitants/km^2^. The rural area, Bavi, is a typical rural village, 60 km west of central Hanoi with agricultural production and livestock breeding as the main economic activities [12].

**Figure 1:**
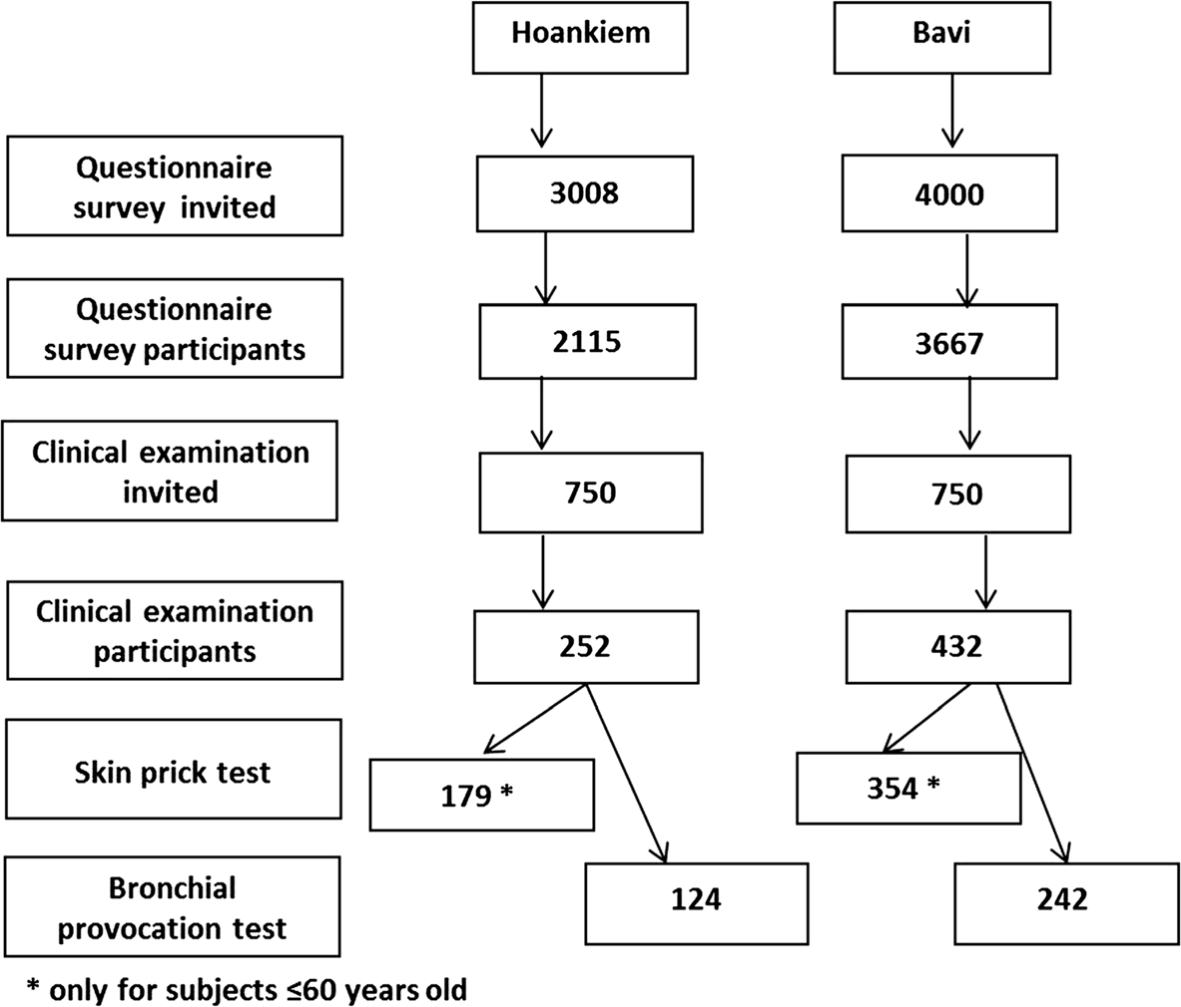
Flow chart of the study design and study population.

Based on a large questionnaire study performed during 2007–09, a random sample of the 5,782 responders was invited to clinical examinations, including skin prick testing (SPT) and interviews [13,14]. To the clinical examinations, 1,500 subjects were invited, 750 from each of the two areas. The age distribution was 23–72 years at the time of examination, and in total 684 (46%) participated. Subjects ≤60 years of age were invited to skin prick tests (SPTs), and 553 subjects participated, mean age 43.5 ± 10.4 years (Figure 1).

### Methods

The clinical study was conducted from March 2009 to April 2010. The research team consisted of trained nurses and medical doctors who performed the data collection including structured interview, lung function test, methacholine challenge, and skin prick test (SPT). All study activities took place at the local commune health care centres. The study was approved by the Medical Ethics Research Committee of Hanoi Medical University. Patient informed consent was obtained before any testing procedures were performed.

#### Questionnaire

The interview questionnaire used was the recently developed Global Allergy and Asthma European Network (GA^2^LEN) questionnaire [15]. Additional questions were taken from the Swedish Obstructive Lung Disease in Northern Sweden (OLIN) questionnaire [16]. The questionnaire included questions mainly about respiratory and allergic symptoms, diagnoses, smoking habits, occupation, and early life exposures.

#### Skin prick test

The SPTs were performed according to the standards developed by the European Academy of Allergology and Clinical Immunology [17] using a lancet with a 1 mm tip on the volar aspect of the forearm. Ten allergen extracts were used; six were provided by ALK, Hörsholm, Denmark: Cat, Dog, *Dermatophagoides pteronyssinus, Dermatophagoides farinae, Alternaria alternata, Cladosporium herbarum,* and four were provided by Stallergens Laboratories, France: Cockroach, *Blomia tropicalis*, mixed grass (cocksfoot, sweet vernal grass, rye grass, meadow grass, timothy) and mixed tree (maple, horse chestnut, plane, false acacia, lime). Histamine 10 mg/ml and glycerol were used as positive and negative controls. A positive reaction was recorded if the mean diameter of the wheal was ≥3 mm. The wheal was recorded after 15 minutes, and the mean wheal diameter was measured by adding the largest diameter and its perpendicular diameter and dividing the sum with two. No subjects had a reaction to the negative control and all subjects had a positive reaction to histamine. The exclusion criteria for participation in SPTs were age >60 years, pregnancy or lactation. The subjects were not allowed to take antihistamines for 3 days prior to the skin prick testing.

#### Lung function and bronchial hyper-reactivity test

The lung function tests were performed using a portable spirometer (Multi-functional spirometer HI-801). Calibration of the spirometer was performed daily and the test procedure followed the ATS recommendations [18]. The reference values were taken from the equation calculated for the East Asia population.

The bronchial hyper-reactivity (BHR) test was performed with methacholine and a nebulizer (Aiolos Systems) following a method developed in Sweden [19]. FEV_1_ after inhalation of saline (0.9%) was used as baseline value. The methacholine concentrations used in the test were 0.125, 0.25, 0.5, 1, 2, 4, and 8 mg/ml. The test was discontinued when FEV_1_ had fallen ≥20% from baseline. The challenge was not conducted if FEV_1_ pre-test was <1.5 L or < 60% of predicted, if the subjects were >60 years, had unstable heart disease or hypertension, was pregnant or lactating, or could not co-operate. In total, 366 subjects participated in the test. Bronchial hyper-reactivity was defined as PC_20_ ≤2 mg/ml.

#### Definitions

*Allergic sensitization*: A positive SPT reaction to any of the tested allergens.

*Ever asthma:* “Have you ever had asthma?”

*Physician-diagnosed asthma:* “Have you been diagnosed as having asthma by a physician?”

*Asthma medicine:* “Are you currently taking any medicine for asthma?”

*Asthma attacks:* “Have you had an attack of asthma in the last 12 months?”

*Asthma hospitalisation:* “Have you ever been hospitalised for asthma?”

*Wheezing last 12 months:* “Have you had wheezing or whistling in your chest at any time in the last 12 months?”

*Recurrent wheeze:* “Do you usually have wheezing, whistling or a noisy sound in your chest when breathing?”

*Allergic rhinitis (AR):* “Do you have nasal allergy including hay fever?”

*AR symptoms last 12 months:* “Have you been troubled by nasal allergies in the last 12 months?”

*Intermittent AR:* (if “yes” to the above question) “Have you been troubled by nasal allergies for more than four days in any one week?”

*Persistent AR:* (if “yes” to the above question) “Did this happen continuously for more than four weeks?”

Definitions of possible risk factors including smoking habits have been described in detail previously [13].

### Analysis

Statistical analyses were performed using PASW version 18.0. Chi-square test, and Fisher’s exact test where appropriate, were used for bi-variate comparison of proportions, and Mantel Haenszel for test for trends. A p-value <0.05 was considered statistically significant. In order to evaluate the representativeness of the participants in the clinical study, the prevalence of symptoms and exposures as reported in the initial questionnaire survey was compared between the participants and the entire cohort [13].

## Results

### Participation and representativeness

Participation was higher in Bavi than in Hoankiem (57.5% vs. 33.6%; p < 0.001). Subjects older than 45 years were more likely to attend than younger subjects (51.5% vs. 39.8%; p < 0.001), as were women compared to men (49.5% vs. 41.7%; p = 0.002). The prevalence of respiratory symptoms, reported asthma and allergic rhinitis were similar among all responders at the initial questionnaire and the subsample who participated in the clinical examinations (Table 1).

**Table 1:** The representativeness of the clinically examined sample based on the questionnaire study data*

**Symptoms or conditions**	**The entire cohort**	**Invited to clinical examinations**	**Participants in clinical examinations**	**Participants in skin prick test**	Difference (p-value)**
	**n = 5,782**	**n = 1,500**	**n = 684**	**n = 533**	
Ever asthma	4.5	4.3	4.1	3.8	0.467
Physician diagnosed asthma	3.9	3.3	3.2	3.0	0.581
Asthma medicine	2.1	1.9	1.6	1.1	0.121
Allergic rhinitis	17.2	20.7	19.2	16.8	0.013
Recurrent wheeze	3.7	3.4	3.7	3.2	0.579
Wheezing without a cold	3.2	2.8	3.4	3.2	0.396
Attack of SOB	5.2	4.9	5.8	4.7	0.433
Dyspnea	3.5	3.6	3.9	4.0	0.458

*Prevalence (%) of symptoms and conditions in the entire cohort, among those invited to the clinical examinations, and those who participated in the clinical examinations.

**Difference between participants in skin prick testing and non-participants in ages ≤60 years old.

### Allergic sensitization

Significantly more men than women were sensitized to both mites (30.9% vs. 21.8%; p = 0.017) and cockroach (16.5% vs. 10.2%; p = 0.033). Among men, 34.1% were sensitized to either mites or cockroach versus 26.1% among women (p = 0.042). The most common sensitizer among both men and women was the storage mite *B. tropicalis* (men 27.7%; women 18.7%; p = 0.013)*,* followed by the house dust mites *D. pteronyssinus* (men 16.5%; women 10.6%; p = 0.045) and *D. farinae* (men 15.3%; women 6.3%; p = 0.001), and cockroach (men 16.5%; women 10.2%; p = 0.033) (Table 2)*.* Sensitization to any animal, any pollen and any mould was considerably lower and contributed with only 2.8 per cent units to the total prevalence of allergic sensitization among men versus 4.9% among women. Smoking habits were not associated with allergic sensitization.

**Table 2:** Prevalence (%) of allergic sensitization by sex, area and age group

**Allergens**	**Sex**			**Area**			**Age**		
	**Male**	**Female**	**Difference, p-value**	**Hoankiem**	**Bavi**	**Difference, p-value**	**≤45 y**	**>45 y**	**Difference, p-value**
	**n = 249**	**n = 284**		**n = 179**	**n = 354**		**n = 278**	**n = 255**	
Cat	5.2	3.9	0.454	5.6	4.0	0.391	5.4	3.5	0.299
Dog	3.6	3.9	0.875	6.1	2.5	0.039	4.7	2.7	0.241
Any animal	8.0	5.6	0.271	9.5	5.4	0.073	7.9	5.5	0.265
Cockroach	16.5	10.2	0.033	11.7	13.8	0.496	15.1	11.0	0.159
*D. pteronyssinus*	16.5	10.6	0.045	19.0	10.5	0.006	16.9	9.4	0.011
*D. farinae*	15.3	6.3	0.001	11.2	10.2	0.721	11.2	9.8	0.612
*B. tropicalis*	27.7	18.7	0.013	22.9	22.9	0.995	27.3	18.0	0.011
Any mite	30.9	21.8	0.017	27.4	25.4	0.628	30.6	21.2	0.014
*A. alternata*	0.8	0.7	0.895	0.6	0.8	0.715	0.7	0.8	0.931
*C. herbarum*	0.4	1.4	0.229	1.7	0.6	0.209	1.1	0.8	0.724
Any mould	1.2	1.8	0.599	2.2	1.1	0.322	1.8	1.2	0.555
Grass pollen	2.8	1.4	0.256	1.7	2.3	0.654	2.5	1.6	0.441
Tree pollen	0.4	1.8	0.138	2.2	0.6	0.084	0.7	1.6	0.353
Any pollen	3.2	2.5	0.602	3.4	2.5	0.594	2.9	2.7	0.926
Any allergen	36.9	31.0	0.146	34.1	33.6	0.915	38.1	29.0	0.026

Sensitization to *D. pteronyssinus* but not *B. tropicalis* was significantly more common in Hoankiem than in Bavi (19.0% vs. 10.5%; p = 0.006), as was sensitization to dog (6.1% vs. 2.5%, p = 0.039). Otherwise no significant difference by area was found (Table 2). In age ≤ 45 years vs. age >45 years, sensitization was more common to *B. tropicalis* (27.3% vs. 18.0%; p = 0.011), *D. pteronyssinus*, (16.9% vs. 9.4%, p = 0.011), any mite (30.6% vs. 21.2%; p = 0.014), and any allergen (38.1% vs. 29.0%; p = 0.026) (Table 2).

**Figure 2:**
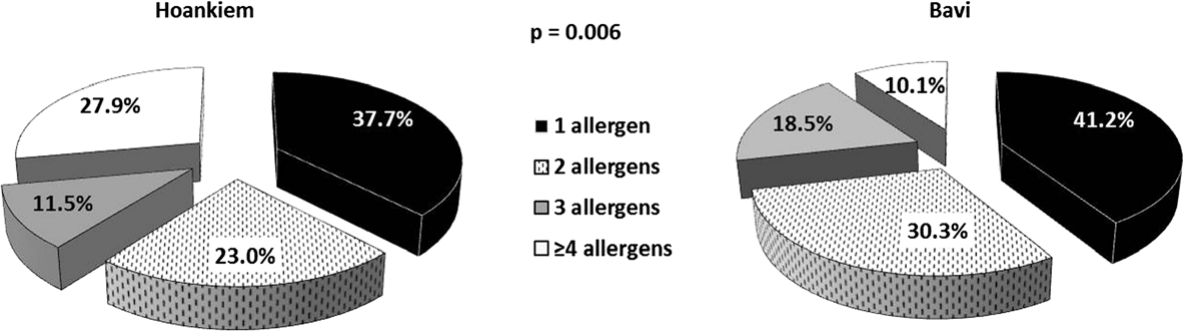
**Proportions (%) of having one, two, three or at least four positive SPT reactions among all sensitized subjects in Hoankiem and Bavi.** Difference (p-value) by study area.

Among the sensitized, 40.0% reacted to a single allergen, while 16.1% was sensitized to ≥4 allergens. Sensitization to ≥4 allergens was significantly more common in the urban area Hoankiem compared to the rural Bavi, p = 0.006 (Figure 2). Similarly, sensitization to ≥4 allergens was more common among men compared to women (p = 0.004), while by age group, i.e. age >45 and ≤45, no difference was found.

**Figure 3:**
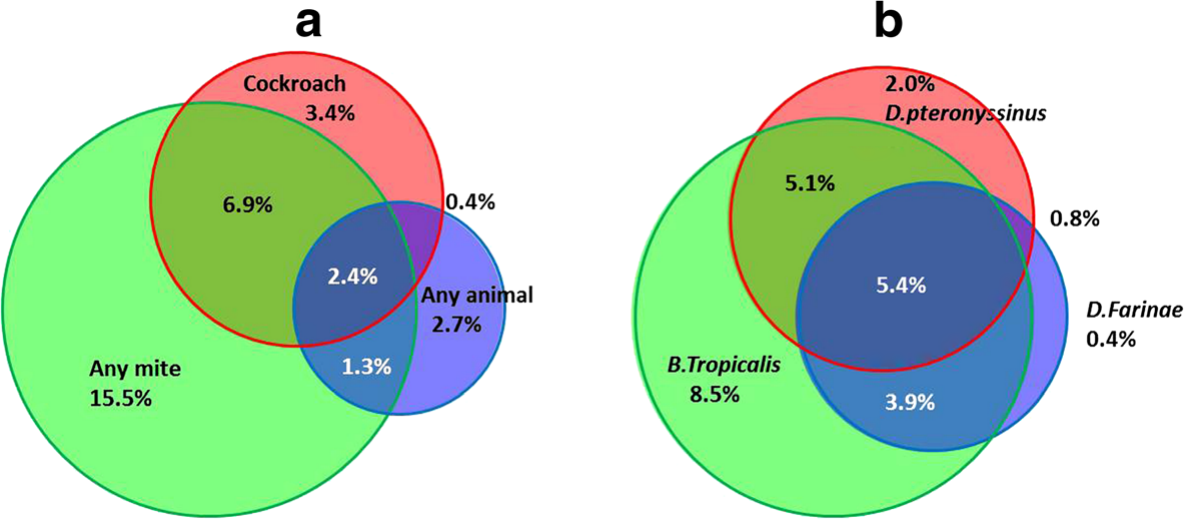
**Proportional Venn diagram. a)** A proportional Venn diagram has been used to quantify the distribution of subjects with a positive SPT to cockroach, any mite, and any animal in the whole population sample. **b)** A proportional Venn diagram has been used to quantify the distribution of subjects with a positive SPT to *B. tropicalis*, *D. pteronyssinus* and *D. farinae* in the whole population sample.

Of all subjects, 2.4% were sensitized to all of the three major allergens groups: cockroach, any mite and any animal (Figure 3a). The association between sensitization to cockroach and mite was strong: 71% of those with a positive SPT to cockroach also had a positive SPT to any mite. Conversely, of mite-sensitized subjects 36% were also sensitized to cockroach, 14% were sensitized also to any animal and 59% were sensitized to mites only. The association between the three mite allergens (*D. pteronyssinus, D. farinae* and *B. tropicalis*) was very strong (Figure 3b). The majority of those sensitized to *D. pteronyssinus* and *D farinae*, respectively, were also sensitised to *B. tropicalis* (79% and 89%, respectively), and only four of the 139 mite-sensitized subjects (0.8% of the population) were sensitized to any of the two house dust mite allergens but not *B. tropicalis*.

### Allergic sensitization in relation to airway diseases and symptoms

Allergic rhinitis (AR) and AR symptoms last 12 months were strongly associated with sensitization to any mite, cockroach, any animal and to any allergen. Persistent allergic rhinitis was significantly associated with sensitization to cockroach, any animal and to any allergen (Table 3). In contrast, ever asthma, physician-diagnosed asthma and the majority of symptoms common in asthma were not more common among subjects sensitized to animals, mites or cockroach, compared to subjects not sensitized to these allergens. However, recurrent wheeze and asthma attacks were positively associated to sensitization to any allergen, p = 0.036 and p = 0.015, respectively.

**Table 3:** Prevalence (%) of symptoms and diseases based on the interview among all subjects and among subjects sensitized (Yes) and not sensitized (No) to common air born allergens

**Symptoms or conditions**	**Prevalence (%)**	**Any animal**			**Any mite**			**Cockroach**			**Any allergens**		
		**Yes**	**No**	p-value^*^	**Yes**	**No**	p-value^*^	**Yes**	**No**	p-value^*^	**Yes**	**No**	p-value*
Ever asthma	6.7	13.9	6.0	0.067	9.4	5.6	0.125	5.7	6.7	0.801	8.9	5.4	0.124
Physician diagnosed asthma	5.0	5.6	5.0	0.892	4.3	5.3	0.635	2.9	5.4	0.396	4.4	5.4	0.636
Asthma medicine	2.5	2.8	2.2	0.827	2.2	2.3	0.928	1.4	2.4	0.615	1.7	2.6	0.513
Asthma attack	2.0	5.6	1.4	0.063	2.9	1.3	0.207	2.9	1.5	0.419	3.3	0.9	0.036
Asthma hospitalization	2.5	2.8	2.0	0.756	2.9	1.8	0.435	0	2.4	0.213	2.2	2.0	0.858
Wheezing last 12 months	24.0	27.8	23.2	0.530	23.7	23.4	0.937	27.1	22.9	0.446	27.8	21.3	0.096
Recurrent wheeze	7.3	8.3	6.3	0.622	10.1	5.1	0.039	11.4	5.6	0.065	10.0	4.5	0.015
Allergic rhinitis	22.4	44.4	21.4	0.001	30.9	20.1	0.009	38.6	20.6	0.001	32.8	17.9	<0.001
AR symptoms last 12 months	19.5	44.4	17.8	<0.001	28.1	16.6	0.003	33.3	17.5	0.002	29.6	14.5	<0.001
Intermittent AR	16.2	25.0	15.7	0.145	23.0	14.0	0.013	21.4	15.6	0.215	22.8	13.0	0.004
Persistent AR	5.1	19.4	4.5	<0.001	7.2	4.9	0.290	14.5	4.1	<0.001	8.4	4.0	0.034

*Difference (p-value) in prevalence of symptoms and diseases among sensitized *v.s* non-sensitized subjects.

The prevalence of allergic rhinitis and AR symptoms last 12 months increased with the number of positive SPT reactions, p < 0.001 each (Table 4). The prevalence of AR symptoms last 12 months was 14.5%, 25.4%, 28.0 and 36.2% in subjects with 0, 1, 2 and ≥3 positive SPTs respectively. Indices of asthma, however, were not associated with increasing number of positive SPT reactions. The prevalence of asthma attacks and recurrent wheeze, which was higher in sensitized compared to non-sensitized subjects, was similar in subjects with 1 positive SPT and subjects with ≥3 positive SPTs (Table 4).

**Table 4:** Prevalence (%) of symptoms and diseases by number of positive SPT reactions

**Symptoms or diseases**	**Number of positive SPT reactions**				
	**0**	**1**	**2**	**≥3**	p-value*
	**(n = 353)**	**(n = 72)**	**(n = 50)**	**(n = 58)**	
Ever asthma	5.4	12.5	6.0	6.9	0.176
Physician-diagnosed asthma	5.4	6.9	2.0	3.4	0.594
Asthma medicine	2.6	0	4.0	1.7	0.463
Asthma attack	0.9	2.8	4.0	3.4	0.197
Asthma hospitalization	2.0	2.8	2.0	1.7	0.973
Wheezing last 12 months	21.3	31.9	24.0	25.9	0.263
Recurrent wheeze	4.5	9.7	10.0	10.3	0.114
Allergic rhinitis	17.9	29.2	28.0	41.4	<0.001
AR symptoms last 12 months	14.5	25.4	28.0	36.2	<0.001
Intermittent AR	13.0	18.1	22.0	29.3	0.010
Persistent AR	4.0	7.1	6.0	12.1	0.080
Bronchial hyper-reactivity, PC_20_ ≤ 2 mg/ml	7.1	10.2	11.4	17.1	0.047

*Difference (p-value) in prevalence of symptoms and diseases by number of positive SPT reactions.

A significant association was found between the number of positive SPT reactions and bronchial hyper-reactivity defined as PC_20_ ≤2 mg/ml (Table 4). Using a PC_20_ of ≤ 4 mg/ml as a cut-off level showed a similar trend but did not reach statistical significance.

## Discussion

This was the first study of allergic sensitization and its association with airway conditions among adults in northern Vietnam. The study is one of few in South-East Asia to include storage mite, which we found to be the most common sensitizer in both urban and rural areas. In addition, the vast majority of those sensitized to house dust mite were also sensitized to storage mite. Sensitization, including mite sensitization, was a strong predictor of rhinitis but not of asthma. Likewise, multiple sensitizations were associated with the prevalence of rhinitis but not indices of asthma. However, the prevalence of bronchial hyper-responsiveness increased with increasing number of positive SPTs.

In this population of North Vietnamese adults, mite and cockroach were the most prevalent sensitizing allergens, similar to previous studies of children and adolescents in southern Vietnam [8,9] and in China [20]. This sensitization pattern has also been demonstrated in adult asthmatics in Singapore and Malaysia [4,5], and our study extends these findings to a general population of adults in South-East Asia. The high temperature and humidity in South-East Asia is an ideal habitat for mites and cockroaches. Although levels of mite and cockroach allergens are not known, the consistently high prevalence of sensitization identifies mite and cockroaches as particularly important sources of indoor allergen in South-East Asia.

Importantly, we identified the storage mite *Blomia tropicalis* as the dominant sensitizing allergen in this general population. This allergen has only been included in a minority of studies, including one of adult asthmatics in Singapore, which also found a high prevalence [4]. *B. tropicalis* typically occurs in stored grain in tropical or subtropical climates. In our study, the prevalence of sensitization to *B. tropicalis* was similar in the urban and rural study areas, suggesting that it may be ubiquitous in Vietnam and possibly in other regions of South- East Asia as well. Sensitization to the house dust mites *D. pteronyssinus* and *D. farinae* was also common, however few subjects were sensitized to house dust mites without concomitant sensitization to *B. tropicalis*. Conversely, 8.5% were sensitized only to *B. tropicalis*, corresponding to one fourth of sensitized subjects. Cross reactivity may contribute, however, cross reactivity between allergen from *B. tropicalis* and *D. pteronyssinus* has previously been shown to be low to moderate [21]. Our findings thus demonstrate that studies in this area which do not include *B. tropicalis* in the allergen panel may substantially underestimate the prevalence of sensitization.

Sensitization to cockroach was less common in our study compared to children in southern Vietnam, despite the similarities in house dust mite sensitization [9]. The lower prevalence of sensitization to cockroach in our study could partly be due to use of a different cockroach allergen extract. This is supported by the relatively low level of sensitization to cockroach in a study in southern Vietnam using the same cockroach species as in our study (*Blatella germanica*) [9]. Also, cockroach sensitization is independently associated with e.g. male sex in our and other studies [22,23] and also with low socioeconomic status [24], and some of the difference between studies could thus be explained by demographic factors.

In large parts of the world mite and cockroach sensitization are typically strong predictors of asthma [24-28], On the contrary, we found no association with physician-diagnosed asthma or the majority of symptoms common in asthma, except recurrent wheeze and asthma attacks. Moreover, the majority of asthma symptoms were equally common in non-sensitized subjects as in subjects with multiple positive SPT reactions, whereas in Western populations, the risk of asthma is typically increased in subjects with multiple sensitizations [6]. However, we identified a moderate positive association of multiple sensitizations with bronchial hyper-responsiveness. Our findings are in line with previous studies in South-East Asia, which generally have found only weak or no associations of allergic sensitization with indices of asthma [2,29]. The reasons for this discrepancy are incompletely known, however the association with hyper-responsiveness but not asthma could to some extent implicate under-diagnosis and poor recognition of asthma symptoms in the general adult population in the study area. This is supported by the relatively low prevalence of reported physician-diagnosed asthma in this area [12]. However, among children in the same area, higher prevalence of asthma has been reported [30].

Unlike asthma, allergic rhinitis was consistently associated with sensitization to mites and cockroach in our study, a finding in line with other studies performed in South-East Asia [2,4,20,31]. The associations were similar for all studied mite species, although co-sensitization between species was common as previously discussed. Its clinical relevance suggests that testing for *B. tropicalis* should not only be used in future studies of sensitization in this region, but also in clinical practice.

Allergic rhinitis was also associated with sensitization to cats or dogs, despite the low prevalence of animal sensitization. In Vietnam, pet keeping inside is uncommon which could contribute to the low level of sensitization seen in our study. Sensitization to dogs was more common in the urban Hoankiem compared the rural Bavi area, which may reflect practices of pet keeping rather than urbanization *per se*, since sensitization in general was equally prevalent in both areas. In general, the recent and rapid urbanization in Vietnam may explain why we did not see a decreased risk of sensitization in subjects raised on farms [10], an otherwise common finding in Western countries [8,32].

The prevalence of sensitization to molds and pollens was very low in our study, results in line with the previously referred study conducted in southern Vietnam [9]. In China, the prevalence of pollinosis varied from 0.5% to 1% in most areas [33]. Also in a study of Thai female high school students only 1.2% was sensitized to molds [2]. In our study, none of the asthma patients were sensitized to molds or pollens, similar to a previous study in Malaysia [5]. Taken together, the data suggest that molds and pollens are allergens of minor importance in South-East Asia.

The population in the studied areas was well-defined and the study sample was randomly selected. Both these facts contribute to the strength of our study and the validity of our results. The response rate in this clinical part of the study was lower, 45.6%, compared to the initial questionnaire survey, 82.5% [14]. The relatively low participation rate might have caused bias. Nevertheless, the prevalence of asthma and respiratory symptoms as reported in the questionnaire study were similar among the participants in the clinical part and in the entire cohort. The prevalence of rhinitis was however somewhat higher among those who attended the clinical examinations. Thus, the modest participation rate in the clinical examinations might not have created any major bias due to non-participation but caused limitations when studying associations.

In conclusion, this study of adults in northern Vietnam identified mite and cockroach as major allergens in rural as well as urban areas. The dominant sensitizer was the storage mite *B. tropicalis*, which should be included in future studies and also in clinical practice, owing to its association with clinical symptoms. Our study adds to the notion that asthma is less associated with sensitization in South-East Asia compared to in the Western world; however the underlying reasons are incompletely known.

## Competing interests

The authors declare that they have no competing interests.

## Authors’ contributions

LHT carried out the studies, participated in the analysis and interpretation of data and drafted the manuscript. EL participated in the analysis and interpretation of data and drafted the manuscript. BA participated in the analysis and interpretation of data and drafted the manuscript. TNV carried out the studies, participated in the analysis and interpretation of data and drafted the manuscript. LB carried out the studies, participated in the analysis and interpretation of data and drafted the manuscript. RE carried out the studies, participated in the analysis and interpretation of data and drafted the manuscript. All authors read and approved the final manuscript.
